# Targeting serine-glycine-one-carbon metabolism as a vulnerability in cancers

**DOI:** 10.1186/s40364-023-00487-4

**Published:** 2023-05-05

**Authors:** Wei Sun, Ruochen Liu, Xinyue Gao, Zini Lin, Hongao Tang, Hongjuan Cui, Erhu Zhao

**Affiliations:** 1 State Key Laboratory of Resource Insects, Medical Research Institute, Southwest University, No.2 Tiansheng Road, Beibei District, 400716 Chongqing, China; 2 Chongqing Engineering and Technology Research Center for Silk Biomaterials and Regenerative Medicine, Chongqing, 400716 China; 3 Engineering Research Center for Cancer Biomedical and Translational Medicine, Southwest University, Chongqing, 400715 China; 4 Jinfeng Laboratory, Chongqing, 401329 China

**Keywords:** Serine-glycine-one-carbon metabolism, Vulnerability, Metabolic enzyme inhibitors, Immunotherapy, Ferroptosis

## Abstract

The serine-glycine-one-carbon (SGOC) metabolic pathway is critical for DNA methylation, histone methylation, and redox homeostasis, in addition to protein, lipid, and nucleotide biosynthesis. The SGOC pathway is a crucial metabolic network in tumorigenesis, wherein the outputs are required for cell survival and proliferation and are particularly likely to be co-opted by aggressive cancers. SGOC metabolism provides an integration point in cell metabolism and is of crucial clinical significance. The mechanism of how this network is regulated is the key to understanding tumor heterogeneity and overcoming the potential mechanism of tumor recurrence. Herein, we review the role of SGOC metabolism in cancer by focusing on key enzymes with tumor-promoting functions and important products with physiological significance in tumorigenesis. In addition, we introduce the ways in which cancer cells acquire and use one-carbon unit, and discuss the recently clarified role of SGOC metabolic enzymes in tumorigenesis and development, as well as their relationship with cancer immunotherapy and ferroptosis. The targeting of SGOC metabolism may be a potential therapeutic strategy to improve clinical outcomes in cancers.

## Introduction

Cell metabolic reprogramming is a common feature of human tumors and refers to the reconnection of cell metabolic flux to produce enough metabolites to support rapid cell proliferation under limited nutrition and stress conditions [1, 2]. Cell growth and proliferation require the construction of new cell components, including proteins, nucleic acids, and lipids, as well as the maintenance of redox, genetic and epigenetic states [3–5]. The metabolic unit known as SGOC metabolism, which provides serine, glycine, one-carbon units and other intermediates, can satisfy many of these requirements [6–8]. Furthermore, SGOC metabolism provides substrates for methylation reactions and affects cellular antioxidative capacity, thus promoting tumor homeostasis [9–11]. In 2014, Mehrmohamadi and his colleagues first proposed the concept of the SGOC metabolic network, and determined its extensive and heterogeneous functions in human cancer [11].

Recent studies have suggested a new role for SGOC metabolism in cancer pathogenesis. In neuroendocrine prostate cancer, SGOC metabolic networks are highly expressed and activated, thus suggesting a targetable vulnerability [12]. In MYCN-amplified neuroblastoma, SGOC metabolism is very active in supplying glucose-derived carbon for serine and glycine synthesis and presents a MYCN-dependent metabolic vulnerability [13–15]. In colorectal cancer (CRC) with ILF3 overexpression, SGOC metabolic enzymes are deregulated under tumorigenic conditions and may be potential targets for cancer therapy [16]. In breast cancer, the SGOC network is a metabolic hallmark inherent to CDK12-induced tumorigenesis, which indicates that an actionable vulnerability exists for breast cancer therapy [17]. Taken together, SGOC metabolism may represent a vulnerability in all highly SGOC-activated tumors in future scenarios. Herein, we summarize the roles of SGOC metabolism in tumorigenesis and development, and discuss their relationship with tumor immunotherapy and ferroptosis. SGOC metabolic enzymes may be potential therapeutic target genes for cancer treatment.

## Serine, glycine and one-carbon metabolism

### Serine and glycine metabolism

Serine is the main donor of one-carbon units, which can enter cells via many different transporter proteins or be synthesized de novo by the cell [18]. Extracellular serine supports the survival and proliferation of many types of cancer cells. A set of metabolite profiles of 60 different cancer cells showed that cancer cells voraciously consume extracellular serine, wherein this consumption ranks second only to that of glutamine among the amino acids [19]. Serine starvation can induce stress and metabolic remodeling, and inhibit cancer progression [4, 20, 21]. Moreover, Yang and colleagues found that tumor protein p53-mediated cell death was significantly enhanced in response to Nutlin-3 treatment during serine starvation [22]. The inhibition of the serine synthesis pathway and dietary serine depletion synergistically inhibit one-carbon metabolism and cancer cell growth [4]. In addition, cancer cells can also obtain serine via lysosomal degradation of proteins, as occurs during macrophage phagocytosis and autophagy [23–25]. The key metabolic enzymes in serine and glycine metabolism include phosphoglycerate dehydrogenase (PHGDH), phosphoserine aminotransferase 1 (PSAT1), phosphoserine phosphatase (PSPH), serine hydroxymethyltransferase 1/2 (SHMT1/2). Serine can be converted to glycine by SHMT1 in the cytoplasm or SHMT2 in the mitochondria [26–28]. During this process, a one-carbon unit separated from serine is transferred to tetrahydrofolate (THF) to produce 5-methyltetrahydrofolate (CH_2_-THF) [29]. CH_2_-THF is a precursor of folate and is reduced to 5-methyltetrahydrofolate (5-CH_3_-THF) by 5,10-methylenetetrahydrofolate reductase (MTHFR); finally, 5-CH_3_-THF is demethylated to yield folate to complete the folate cycle [30].

In addition, many cancer cells contain a glycine cleavage system through which glycine is cleaved in the presence of the glycine decarboxylase complex to produce ammonia, carbon dioxide, and methylenetetrahydrofolate to fuel the production of one-carbon units [31].

### Folate-mediated one-carbon metabolism (FOCM)

**Fig. 1:**
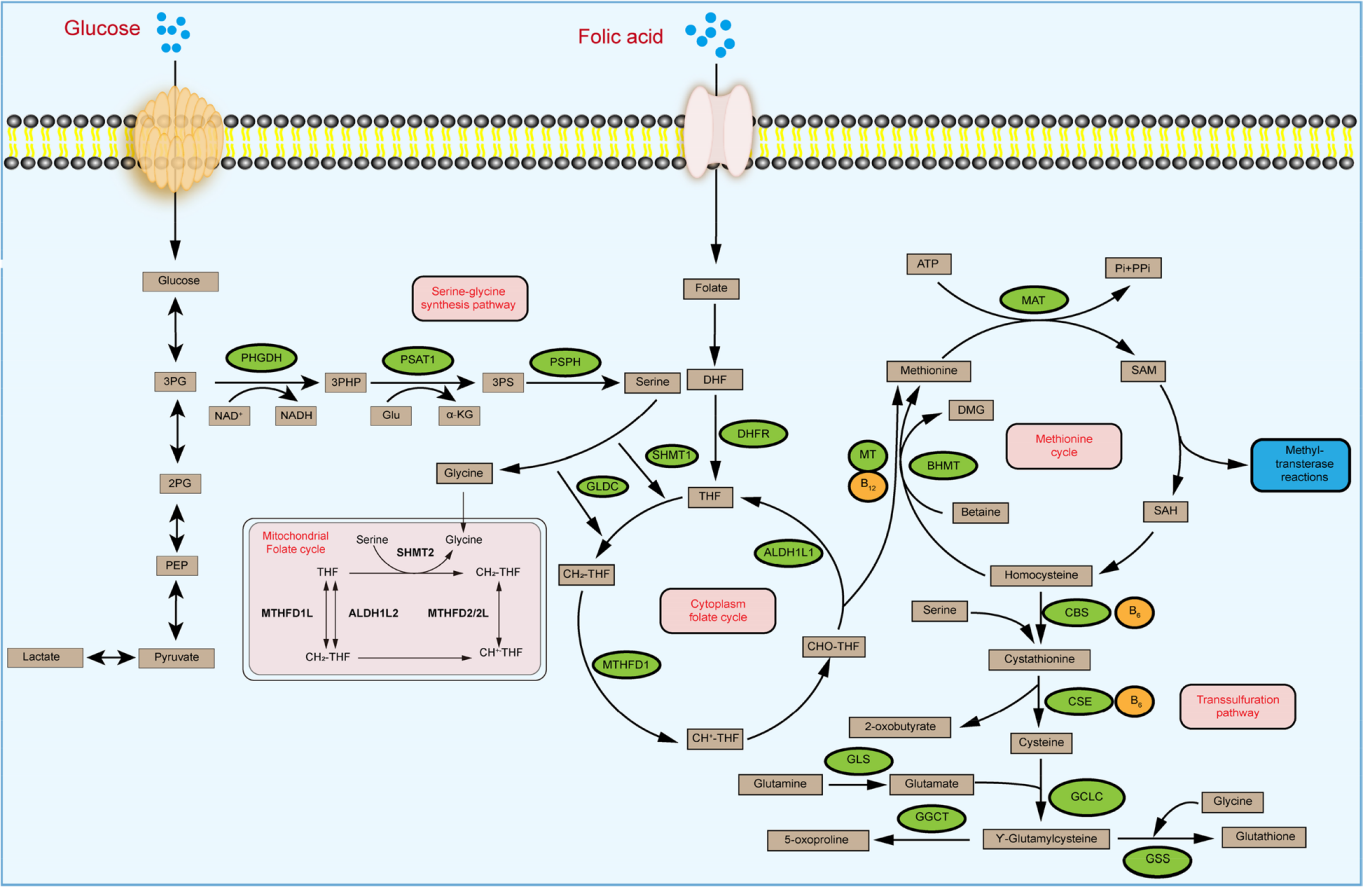
Serine-glycine-one-carbon metabolic pathway

Folic acid is a water-soluble B vitamin that can be converted to THF in vivo and is involved in many biochemical reactions in vivo (Fig. 1). Folate metabolism often occurs in both the cytoplasm and mitochondria and is compartmentalized in distinct regions in the cytoplasm, nucleus and mitochondria, depending on whether the one-carbon units are derived from serine or glycine catabolism [32–34]. The key metabolic enzymes in FOCM metabolism include methylenetetrahydrofolate dehydrogenase 1/1L (MTHFD1/1L), methylenetetrahydrofolate dehydrogenase 2/2L (MTHFD2/2L) and aldehyde dehydrogenase 1 family member L1/L2 (ALDH1L1/2) and so on. In most cultured cells, mitochondrial SHMT2 transfers the β-carbon atom from serine to THF to generate CH_2_-THF. This folic acid intermediate can also be produced by separating a one-carbon unit from glycine in a reaction catalyzed by the glycine cleavage system [35]. Subsequently, MTHFD2 or MTHFD2L uses NAD^+^ or NADP^+^ to oxidize CH_2_-THF to generate 10-formyltetrahydrofolate (10-CHO-THF) and produce a molecule of Nicotinamide adenine dinucleotide phosphate (NADPH) [36, 37]. Moreover, 10-CHO-THF can be used for the formylation of mitochondrial promoters [38, 39]. In addition, it can provide fuel for cytoplasmic and nuclear reactions or be excreted from the cell [40]. Mitochondrial 10-CHO-THF does not cross the mitochondrial membrane; thus, one of the one-carbon units in 10-CHO-THF is converted to formate in an MTHFD1L-mediated reaction, and formate can be exported to the cytoplasm [41]. During this process, adenosine diphosphate (ADP) can be phosphorylated to adenosine triphosphate (ATP) or used to generate THF and release CO_2_ via ALDH1L2, accompanied by NADPH production [42]. The formate transferred to the cytoplasm is dehydrogenated by MTHFD1 in a reaction that consumes ATP to regenerate cytosolic 10-CHO-THF for the de novo synthesis of purines [43]. This reaction can generate cytosolic CH_2_-THF for homocysteine remethylation and thymidylate synthesis via MTHFR or thymidylate synthase (TYMS) [35]. This CH_2_-THF can be reduced to THF via cytoplasmic SHMT1, which completes the folate cycle and the conversion of glycine to serine [44]. In addition, folic acid is reduced to 7,8-dihydrofolate (DHF) and then to THF by dihydrofolate reductase (DHFR) [30]. In conclusion, the folic acid cycle, as the common metabolic pathway between SGOC and one-carbon metabolism, has high plasticity.

### Methionine cycle and transsulfuration pathway

The methionine cycle is commonly used to produce S-adenosyl-l-methionine (SAM), which is an ubiquitous methyl donor used by a large class of SAM-dependent methyltransferases for DNA, RNA, protein and lipid methylation [45]. The SGOC pathway is interconnected with the methionine cycle through the action of MTHFR, which catalyzes the irreversible conversion of CH_2_-THF to 5-CH_3_-THF. Afterwards, 5-CH_3_-THF is used by ubiquitously expressed methionine synthase (MS) to remethylate homocysteine in a vitamin B12-dependent reaction [46]. Furthermore, serine apparently plays an important role in the methionine cycle in vivo, and stable isotope tracing studies have shown that most methyl groups used for systemic homocysteine remethylation are derived from serine [47]. However, some reports have shown that serine-derived one-carbon units cannot be used to support remethylation when exogenous methionine levels are high [48, 49]. Glutathione (GSH) is synthesized from cysteine, glutamate and glycine in cytoplasmic lysates and can be transported to various cellular compartments [50]. Serine and homocysteine are also linked to the methionine cycle via the transsulfuration pathway, and both homocysteine and serine are precursors of cysteine synthesis. Due to the fact that both glycine and cysteine are products of serine metabolism, the depletion of serine results in lower GSH levels, whereas the activation of the SGOC pathway increases GSH synthesis [51]. In conclusion, the SGOC pathway is closely associated with the methionine cycle and transsulfuration pathway.

## Serine-glycine-one-carbon metabolism in tumors

The SGOC network is a metabolic hallmark that is frequently upregulated in tumors and orchestrates two nearly identical, intertwined methylation cycles in the cytoplasm and mitochondria, thus having high clinical relevance [18, 52, 53]. The key metabolic enzymes in SGOC (folate cycle) metabolism include PHGDH, PSAT1, PSPH, SHMT1/2, MTHFD1/1L and MTHFD2/2L. Recently, an increasing number of studies have reported that SGOC metabolic enzymes are highly expressed in various cancers and indicate poor prognosis. PHGDH is the major rate-limiting enzyme in the first step of the SGOC pathway, which is abnormal in various diseases, especially in cancers [54, 55]. The expression of PHGDH in pancreatic cancer patients is related to tumor size, lymph node metastasis, and TNM stage of pancreatic cancer patients; in addition, it is an independent prognostic indicator [56]. In lung cancer, SHMT1 and SHMT2 are both highly associated with the infiltration of different types of immune cells, and are potential prognostic biomarkers [57, 58]. A study of 7,309 patients with non-Hodgkin’s lymphoma showed that the SHMT1 C1420T polymorphism may be associated with the risk of developing non-Hodgkin’s lymphoma [59]. These data mentioned above suggest that SGCO metabolic enzymes may be a marker of tumor prognosis.

### Redox effect of SGOC in tumors

Recent studies have demonstrated the effects of SGOC metabolism on dynamic redox balance and epigenetics [48]. The redox state is mainly determined by the dynamic balance between the generation of reactive oxygen species (ROS) and the activation of the antioxidant system [60]. SGOC-related metabolic enzymes can affect the NADPH/NADP^+^ ratio in tumors and regulate the redox state of cells. In fact, genomic analyses have shown that many cancers, especially breast cancer and non-small cell lung cancer (NSCLC), exhibit amplification and upregulated expression of SGOC metabolic enzymes, such as PHGDH and SHMT2 [61, 62]. Research has shown that the serine catabolic enzyme SHMT2, is induced when MYC-transformed cells are subjected to hypoxia; in the mitochondria, SHMT2 can initiate serine degradation to CO_2_ and NH4^+^, resulting in the net production of NADPH from NADP^+^ [63]. Knockdown of SHMT2 in MYC-dependent cells reduced the cellular NADPH:NADP^+^ ratio, increased cellular reactive oxygen species, and triggered hypoxia-induced cell death [63, 64]. In addition, one-carbon units for purine and thymidine synthesis can be generated from serine by cytosolic or mitochondrial folate metabolism [40]. Mitochondrial folate metabolic enzymes play a crucial role in this process. Folate metabolism can produce mitochondrial NADPH through ALDH1L2 and potential MTHFD2, and the knockdown of SHMT2 in some cancer cell lines increases their vulnerability to oxidative stressors [65]. The NADPH/NADP^+^ ratio in turn may play an important role in regulating the cytosolic flux of one-carbon units through the MTHFD1 dehydrogenase reaction [66].

### Epigenetic roles of SGOC in tumors

Histone methyltransferase G9A promotes the transcription of key SGOC metabolic enzymes by maintaining an active chromatin state marked by histone H3 lysine 9 monomethylation (H3K9me1) in an ATF4-dependent manner [67]. SUMOylation is a reversible post-translational modification by conjugating with small ubiquitin-like modifiers (SUMOs) and a common protein modification in cancers [68]. NRF2 SUMOylation promotes the elimination of ROS in cells by increasing the transcription of glutathione peroxidase 2 (Gpx2), which leads to the upregulation of PHGDH in hepatocellular carcinoma (HCC) cells. These changes promote the production of one-carbon units in the de novo synthesis of serine and purine, thus promoting HCC [69]. Researchers have found that KDM4C epigenetically activates pathway genes under steady-state and serine deprivation conditions by removing the repressive histone modification histone H3 lysine 9 trimethylation (H3K9me3), in the serine-glycine synthesis pathway [70]. This finding links KDM4C-mediated H3K9 demethylation and ATF4-mediated transactivation in amino acid metabolism reprogramming for cancer cell proliferation. The deprivation of the SGOC metabolic pathway can lead to a significant drop in total ATP levels in rapidly proliferating cells, thus reducing the transfer of methyl to DNA and RNA, which can lead to changes in methyl transfer but will not induce the activation of AMP activated protein kinase (AMPK) [48]. One study showed that SHMT2 desuccinylation is a key signal for cancer cells to adapt to the serine metabolism process to achieve rapid growth, and the authors emphasized that SIRT5, as a candidate target to inhibit serine catabolism, is a strategy to block tumor growth [71].

### Transcriptional regulation of SGOC in tumors

In addition to the effects of SGOC metabolism on dynamic redox balance and epigenetics, all of the SGOC metabolic enzymes are transcriptionally regulated by various transcription factors during the stress response or oncogene activation [72]. Ma et al. identified interacting proteins and detected their regulatory effects on translation initiation [73]. They found that PHGDH not only catalyzes serine synthesis and activates the AKT pathway but also interacts with the translation initiation factors eIF4A1 and eIF4E to promote the assembly of eIF4F on the 5’mRNA structure to increase the expression of related proteins, thus promoting the development of pancreatic cancer [73]. Studies have shown that in the absence of amino acids, cancer cells induce the expression of PHGDH, PSAT1, and PSPH in a GCN2-ATF4-dependent manner to produce sufficient amino acids [70, 74, 75]. Other transcription factors such as NRF2 and MYC, can also activate SGOC metabolism [69, 76, 77]. There is a MYC binding site E-box at the PHGDH, PSAT1, and SHMT gene sites, and knockout of MYC reduces their expression [77, 78]. In addition, HIF-1 and HIF-2 transcription factors can induce the expression of PHGDH, PSAT1, and SHMTs in breast cancer cell lines under hypoxia [79, 80]. The transcription regulators TAZ and YAP (TAZ/YAP) have become tumor-promoting factors that drive many carcinogenic characteristics, including improving cell growth, resisting cell death, and promoting cell migration and invasion. TAZ/YAP can induce the expression of glutamate oxaloacetate transaminase 1 (GOT1) and PSAT1 to produce more α-ketoglutarate and to promote the growth of breast cancer cells [81]. Recently, Liu et al. found that the lysine 64 residue (SHMT2K64) on SHMT2 and β-catenin and the transcription factor TCF4 interact to form SHMT2/β-catenin/TCF4 positive feedback loop, which inhibits ubiquitination-mediated degradation of β-catenin and promotes the proliferation and metastasis of CRC cells [29].

In conclusion, enhanced SGOC pathway activity may affect cancer cell processes, especially metabolism. Metabolites in the SGOC pathway (synthetic precursors of macromolecules, reducing substances, etc.) meet the metabolic requirements of rapid growth and proliferation of cancer cells. Moreover, the targeting of SGOC metabolic enzymes undoubtedly provides a new direction for exploring tumor therapy and brings hope for further research on tumor therapy.

## Serine-glycine-one-carbon metabolism in cancer immunotherapy

The immune system plays an important role in controlling cancer progression. From the perspective of oncogenesis, tumor cells are transformed from normal cells, and this process from “self” to “non-self” is often closely monitored by the immune system and affected by an effective immune response [82]. The innate and adaptive immune systems interact to achieve effective anti-tumor immune monitoring [83]. Cancer immunotherapy has changed the cancer treatment paradigm, and these therapies aim to improve the anti-tumor immune response [84]. T lymphocytes are sentinels of the adaptive immune system, which are specifically used to identify and eliminate threats to the host [85]. The demand for specific nutrients that support the function of T cells increases the possibility that the metabolic microenvironment and availability of nutrients affect immunity by affecting the function of T cells [86]. Recently, some researchers have reported on the key role of non-essential amino acid serine in the effector T-cell response. Serine is essential for many biosynthetic and signal transduction pathways, including protein, nucleotide and glutathione synthesis, and is crucial for the growth and survival of proliferating cells [87]. After T-lymphocyte activation, T cells upregulate the enzymes of the SGOC metabolic network, and rapidly increase the process of serine conversion to one-carbon metabolism [88]. From the perspective of mechanism, serine provides glycine and one-carbon unit for de novo synthesis of proliferating T cells, and one-carbon unit in formate can rescue T cells from serine deficiency [88]. This suggests that the availability of serine in vivo may have important therapeutic significance for the immunotherapy and anti-tumor T-cell responses. Folic acid dependent one-carbon metabolism is a key metabolic process supporting cell proliferation, thus providing a carbon source for the synthesis of nucleotides in DNA and RNA [89]. Luteijn et al. determined that SLC19A1, as a folic acid organophosphorus reverse transporter, is the main transporter of cyclic dinucleotides (CDNs) by using genome wide CRISPR interference screening technology [90]. The inhibition of SLC19A1 can reduce the transport of folic acid, thereby reducing the uptake of CDNs by cancer cells [90, 91]. This discovery is of great significance for cancer immunotherapy and the host’s responsiveness to pathogenic microorganisms that produce CDNs. Researchers have also found that an immunosuppressive subset of tumor cells can be distinguished from the nonimmunosuppressive population by its upregulation of folate receptor beta (FRβ) and restriction to immunosuppressive tumor microenvironment [92]. Pemetrexed, which is a folate pathway inhibitor, can increase the activation of T cells in mouse tumors, and effectively induce immunogenic cell death in mouse tumor cells, as well as exert the inherent effects of T cells in vitro, such as enhancing mitochondrial function and T-cell activation [93]. Interestingly, some researchers have found that tryptophan (rather than serine) is the theoretical source of IDO1 (an enzyme in tumor immune escape) metabolism of one-carbon unit, and their research results showed that when cancer cells express IDO1, it will promote tryptophan to generate a carbon unit for the de novo synthesis of purine nucleotides [94]. Under the condition of low serine, tryptophan can be used as an alternative carbon source to support proliferation [95, 96]. Cancer cells release tryptophan derived formate, which can be used by pancreatic stellate cells to support the synthesis of purine nucleotides, thus avoiding the use of immunotherapy [95].

Recently, it has been reported that PSAT1 hypermethylation is related to T-cell dysfunction, shortened survival time and immune cell infiltration in breast cancer [97]. In addition, the expression of PSAT1 in lung cancer was significantly positively correlated with tumor mutational burden, and negatively correlated with tumor immune dysfunction and rejection [97]. It has been suggested that PSAT1 may be a new biomarker for the survival of lung cancer patients and for predicting the efficacy of immunotherapy. The infiltrating immune cells in ferroptosis-related genes (FRGs) in gastric cancer samples of the TCGA-STAD dataset were estimated by using the CIBERSORT and XCELL algorithms [98]. It was found that the overexpression of Hub FRGs (*MYB, PSAT1, TP53 and LONP1*) were positively correlated with the infiltration of activated CD4^+^ T cells, especially Th cells [98]. It is suggested that effective intervention on Hub FRGs is helpful to promote the activation of CD4^+^ T cells in patients with GC and to improve the efficacy of immunotherapy. In addition, researchers have found that the high expression of another key enzyme of SGOC metabolism (PSPH) was negatively correlated with CD8^+^ T cells, macrophages, and neutrophils, thus affecting the survival of patients with neuroblastoma [99]. Similarly, SHMT2 was significantly associated with CD8^+^ T cell infiltrates and highly expressed in breast, liver and lung cancer, and kidney renal papillary cell carcinoma [100]. These data suggested that PSPH and SHMT2 may be a promising indicator of the prognosis and cancer immunotherapy by affecting the infiltration level of immune cells. In bladder cancer, high expression of MTHFD2 was associated with PD‑L1 activation via the PI3K/AKT signaling pathway, suggesting that it could be a promising marker of cancer immunotherapy [101].

## Serine-glycine-one-carbon metabolism in ferroptosis

**Fig. 2:**
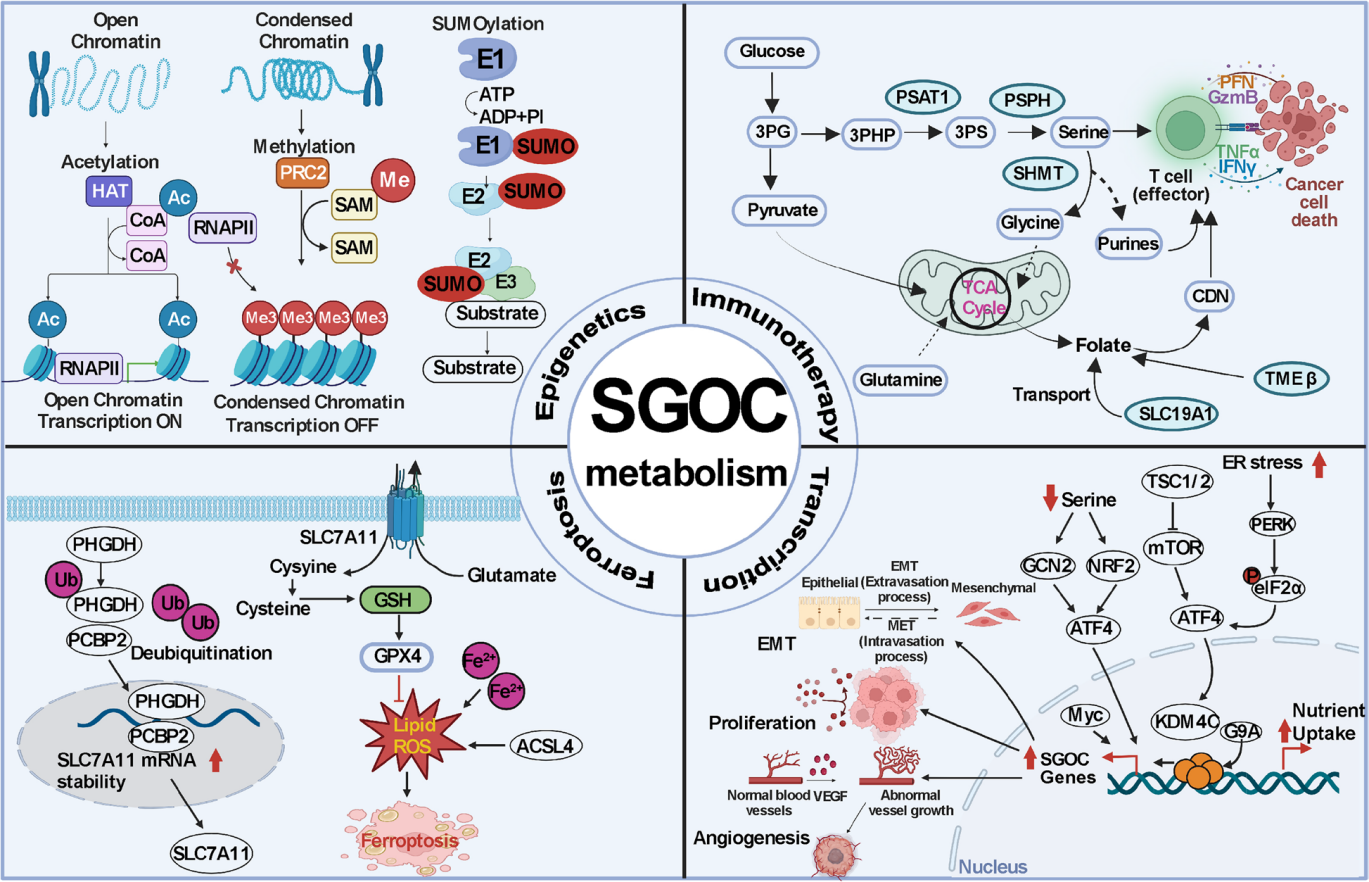
The roles of serine-glycine-one-carbon metabolism in tumors

Ferroptosis is a newly discovered form of programmed cell death, that is the result of excessive oxidation of iron-dependent polyunsaturated fatty acids [102, 103]. The three key characteristics of ferroptosis include membrane lipid peroxidation, availability of intracellular iron, and loss of antioxidant defense [104]. PHGDH, as the first rate-limiting enzyme of the SGOC metabolic pathway, plays an important role in ferroptosis-related pathways. Researchers have found that PHGDH can bind to the RNA-binding protein PCBP2 and inhibit its ubiquitination degradation; subsequently, PCBP2 stabilized SLC7A11 mRNA and increased its expression, thus inhibiting ferroptosis (Fig. 2) [105]. In gastric cancer, PSAT1 was identified as a ferroptosis-related gene and a new potential biomarker, papillary renal cell carcinoma and amyotrophic lateral sclerosis [98, 106, 107]. In liver cancer, the overexpression of c-Jun can activate the transcription of PSAT1 by directly binding with its promoter region, thereby antagonizing the ferroptosis induced by erastin [108]. In triple-negative breast cancer, MTHFD2 was identified as a ferroptosis regulator and prognostic biomarker [109].

Small molecular substances or metabolic pathways are also recognized as important influencing factors of ferroptosis. The amino acid glutamine was identified as the inducers of ferroptosis; and the glutamine-fueled intracellular metabolic pathway, glutaminolysis, was identified as the essential component of ferroptosis [110]. Homocysteine is an amino acid involved in gene methylation and can be generated by the SGOC metabolic pathway [111]. It was found that homocysteine promotes a degenerative cell phenotype (involving increased oxidative stress and cell death by ferroptosis) mediated by upregulated methylation of GPX4 [112].

## Serine-glycine-one-carbon metabolism and noncoding RNAs

### PSAT1 and noncoding RNAs

**Fig. 3:**
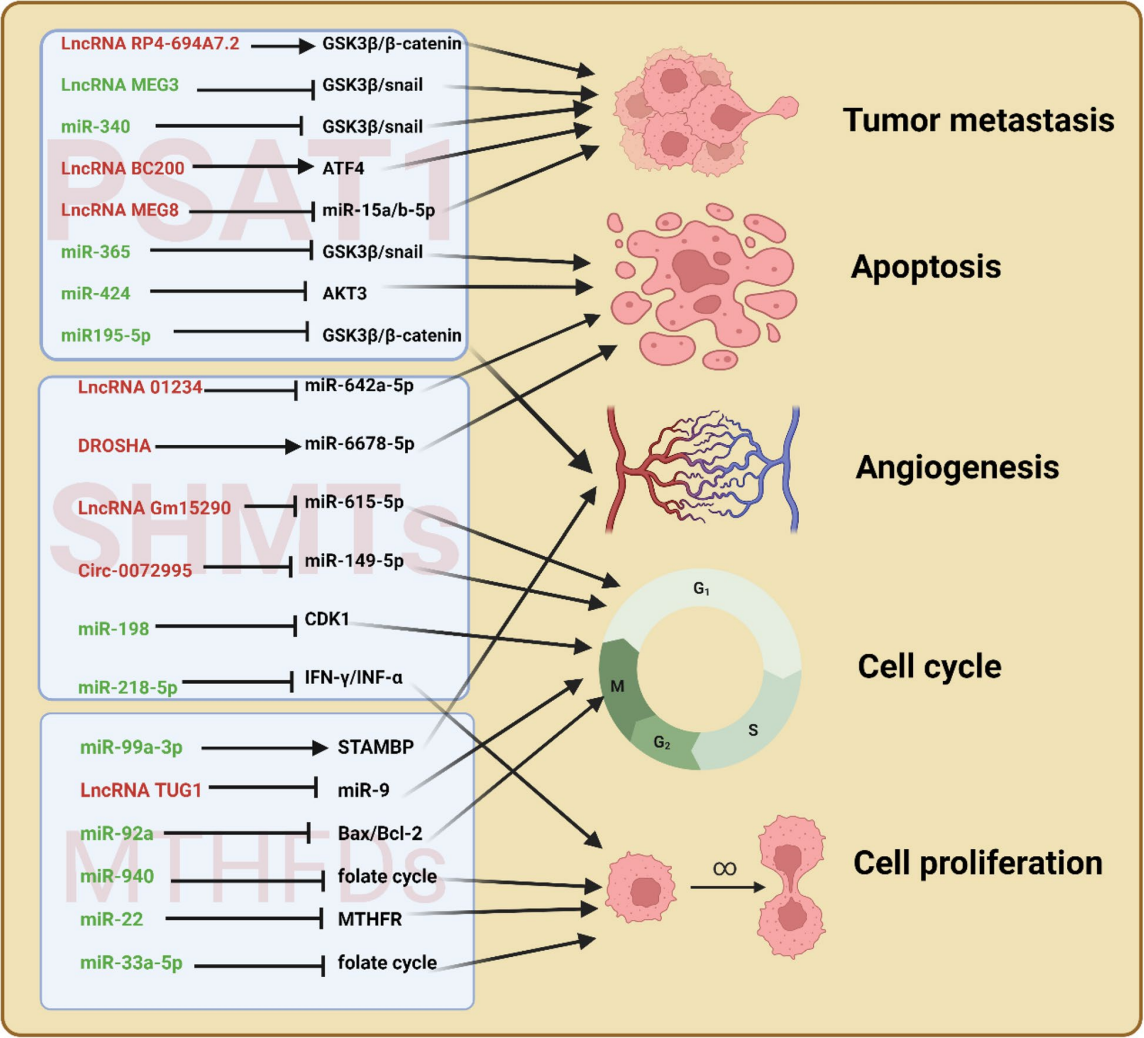
The SGOC metabolic enzymes and regulatory non-coding RNA

Long noncoding RNAs (lncRNAs) can epigenetically regulate gene expression and cellular signaling pathways in different types of cancers [113, 114]. Accumulating evidence shows that lncRNAs are interlinked with PSAT1 and play a major role in cancer cell proliferation, angiogenesis and invasion [114–116]. The lncRNA RP4-694A7.2 levels in HCC tissues are higher than those in normal liver tissues and RP4-694A7.2 is also highly expressed in HCC cell lines [114]. RP4-694A7.2 regulates the glycolytic function of PSAT1 during HCC cell growth and invasion via the GSK3β/β-catenin pathway [114]. Interestingly, lncRNA MEG3 exerts tumor-suppressive effects and inhibits Epithelial-mesenchymal transition (EMT) by suppressing the PSAT1-dependent GSK3β/Snail signaling pathway in esophageal squamous cell carcinoma (ESCC) [115]. In NSCLC, the lncRNA MEG8 is expressed at higher levels in tumor tissues than in normal adjacent tissues and promotes tumor progression by regulating the miR-15a/b-5p/PSAT1 axis [117]. In addition, the lncRNA BC200 promotes the migration and invasion of cancer cells via the regulation of ATF4 expression, which in turn regulates the expression of PSAT1 in ESCC [116] (Fig. 3).

Abnormal expression of miRNAs plays an important role in the development of various cancers. In ESCC, the expression of miR-340 is negatively correlated with that of PSAT1 and significantly lower in tumor tissues than in paraneoplastic tissues [118]. Moreover, the high expression of miR-365 inhibits cell proliferation, invasion, colony formation and EMT and these inhibitions are reversed by the overexpression of PSAT1 [119]. In CRC, miR-424 directly inhibits PSAT1 expression at the transcriptional level, thereby supressing cell proliferation and inducing apoptosis [120]. In ovarian cancer, the overexpression of miR-195-5p reduces cisplatin resistance and angiogenesis by inhibiting PSAT1-dependent GSK3β/β-catenin [121]. Interestingly, miR-15a/b-5p is also regulated by the lncRNA MEG8 and affects the proliferation, migration and invasion of NSCLC cells through the downregulation of PSAT1 expression [117]. In summary, PSAT1 may provide a more effective therapeutic strategy for cancer treatment.

### SHMT and noncoding RNAs

The overexpression of the lncRNA Gm15290 exerts tumor-stimulating effects through the inhibition of miR-615-5p, which targets the genes insulin-like growth factor 2 (IGF2), AKT2, and SHMT2 [122, 123]. In colon cancer, LINC01234 is highly expressed and acts as a competing endogenous RNA for miR-642a-5p, which targets SHMT2 [124]. Furthermore, miR-6778-5p positively regulates SHMT1, thus mediating compensatory activation of cytoplasmic one-carbon metabolism, which plays an essential role in the maintenance of gastric cancer stem cells (GCSCs) [125]. The deletion of miR-6778-5p or SHMT1 significantly reduces GCSC sphere formation and increases 5-FU sensitivity in Drosha-knockdown cells [125]. In lung cancer, miR-198 inhibits cell proliferation in vitro and in vivo by directly targeting SHMT1 [126]. In addition, miR-218-5p suppresses the cytotoxic effect of natural killer cells by targeting SHMT1 in lung cancer [127]. In addition, the circRNA circ_0072995 was demonstrated to promote a malignant phenotype and anaerobic glycolysis by competitively binding miR-149-5p to upregulate its downstream gene SHMT2 in breast cancer [128].

### MTHFD2 and noncoding RNAs

MTHFD2 has been confirmed to be a target gene of miR-33a-5p that suppresses CRC cell growth by inhibiting MTHFD2 [129]. In acute myeloid leukemia (AML), miR-92a inhibits cell proliferation and induces apoptosis by directly suppressing MTHFD2 expression [130]. The high-expression of miR-504-3p is associated with good prognosis in AML patients and may serve as a tumor suppressor by targeting MTHFD2 [131]. In breast cancer, miR-9 exerts anti-proliferative, anti-invasive and proapoptotic effects by targeting MTHFD2 [132]. Interestingly, the lncRNA taurine upregulated gene 1 (TUG1) was found to negatively regulate miR-9 expression but positively regulate the expression of MTHFD2 in breast cancer cells [133]. In glioma, miR-940 suppresses tumor progression by inhibiting mitochondrial folate metabolism, which directly targets MTHFD2 [134]. In gastric cancer, miR-22 inhibits cell proliferation by inducing a deficiency in endogenous SAM by reducing MTHFD2 and MTHFR expression [135]. In head and neck squamous cell carcinoma (HNSCC), low expression of miR-99a-3p, which targets MTHFD2, significantly predicts poor prognosis [136]. These results indicate that miRNAs targeting MTHFD2 regulate tumor progression and may be new biomarkers.

Red font, Promoting the expression of targeted SGOC metabolic enzymes; Green font, Inhibiting the expression of targeted SGOC-metabolic enzymes.

## Therapeutic targeting of serine-glycine-one-carbon metabolic enzymes

### Inhibitors of enzymes in serine-glycine biosynthesis

PHGDH inhibitors can be divided into two main types: synthetic and natural chemicals (Table 1). The synthetic chemicals include BI-4924, CBR-5884, NCT-503, PKUMDL-WQ-2201 and so on [165, 138]. The natural chemicals include azacoccone E and ixocarpalactone A [141, 142] Researchers have found that the most effective PSPH inhibitor using L-phosphoserine as the substrate is p-chloromercuriphenylsulfonic acid (CMPSA), followed by Glyceryl phosphocholine [144]. Moreover, clofazimine is a specific inhibitor of PSPH [143]; D-AP3 is selective and is the most effective competitive inhibitor of PSPH [166]. As an SHMT inhibitor, the compound SHIN1 with the pyrazolopyran scaffold exerts potent and specific on target activity against SHMTs [36]. In addition to having selective activity against SHMT1, compound 2.12 also displayed anticancer activity in the mid-micromolar range [147]. AGF347, a folate mimetic, exerts significant in vivo anti-tumor effect, providing the candidates for therapeutic targeting of SHMTs [146].

**Table 1:** Small-molecule inhibitors of SGOC metabolism

Target enzymes	Inhibitor	Structure	Comments	Indication/most advanced clinical phase	Refs
PHGDH	BI-4924		Competitive inhibitor	Experimentala and cancer/preclinical	[137]
PHGDH	CBR-5884		Inhibition of de novo serine synthesis	Experimentala and cancer/preclinical	[138]
PHGDH	NCT-503		Inhibition of de novo serine synthesis	Experimentala and cancer/preclinical	[139]
PHGDH	PKUMDL-WQ-2201		PHGDH allosteric inhibitor	Experimentala and cancer/preclinical	[140]
PHGDH	Azacoccone E		Non-competitive inhibitor	Cancer and other diseases /preclinical	[141]
PHGDH	Ixocarpalactone A		PHGDH allosteric inhibitor	Cancer and other diseases /preclinical	[142]
PSPH	Clofazimine		Competitive inhibitor	FDA approved treatment for leprosy and tuberculosis	[143]
PSPH	Glycerophosphorylcholine		Non-competitive inhibitor	Clinical Trials, Dementia/preclinical	[144]
SHMTs	SHIN1		Inhibition of glycine and CH_2_-THF generation	Cancer and other diseases /preclinical	[145]
SHMT1/2 GART	AGF347		Inhibition of glycine and CH_2_-THF generation	NSCLC, colon, Pancreatic/ preclinical	[146]
SHMT1/2	2.12		Competitive inhibitor	Lung cancer/ preclinical	[147]
MTHFD2	LY345899		Induction of apoptosis through reduced mitochondrial NADP(H) generation	Colorectal cancer/preclinical	[148]
MTHFD1/2	Carolacton		Inhibition of both substrate and cofactor binding in active site	Colon, endocervical cancer cell/preclinical	[149]
TYMS DHFR GART	Pemetrexed		Inhibition of cell proliferation by limiting thymidylate for DNA synthesis	Various solid and hematological tumors/Approved	[150]
TYMS, DHFR	Methotrexate		Induction of cell death by depleting THF levels	FDA- approved for rtheumatoid arthritis, and neoplastic diseases Osteosarcoma Phase II	[151]
TYMS	5-FU		Inhibition of DNA synthesis by blocking conversion of dUMP to dTMP	Various solid and hematological tumors/Approved	[152]
FOLR1	Farletuzumab	Monoclonal antibody of IgG1κ	Competitive inhibitor	Ovarian cancer/ Phase I Phase II Phase II	[153–155]
DHFR	Trimetrexate		Inhibition of the production of DNA and RNA precursors and lead to cell death	Bacterial infection、cancers/ Phase II	[156]
DHFR/ TYMS	Raltitrexed		Reduction of dTMP level, and increase of dUMP level	Approved by European Medicines Agency (EMA) to treat colorectal cancer/Phase IV HNSCC Phase IV	[157]
DHFR	Piritrexim		Direct inhibition of DNA incorporation of deoxyuridine nucleoside	Bladder cancer/Phase II	[158]
TYMS	ZD-9331		A water-soluble non-polyglutamatable TYMS inhibitor	Ovarian cancer/Phase II	[159]
TYMS	GS7904L		Inhibition of DNA synthesis by blocking conversion of dUMP to dTMP	Colorectal cancer/Phase I, HNSCC/Phase II, Advanced Solid Tumors/Phase I, Gastric cancer /Phase II, Locally Advanced or Metastatic Adenoma of the Biliary Tract/Phase II	[160]
TYMS	ONX-0801		A cyclopenta[g]quinazoline-based inhibitor	Advanced Solid Tumors /Phase I	[161]
MAT2A	AG-270		Allosteric inhibitors	Advanced Solid Tumors and Lymphoma/Phase I	[162]
GART	Lometrexol		Inhibition of de novo purine synthesis	Lung Cancer/Phase I	[163]
CBS/CSE	aminooxyacetic acid		Inhibition of aspartate aminotransferase activity	Experimentala and cancer/preclinical	[164]

### Inhibitors of enzymes in one-carbon metabolism

One-carbon metabolism supports vital events for the growth and survival of proliferating cells whose enzymes are associated with cancer progression [72, 100, 167]. Aminopterin is an anti-folate drug that has been found to relieve childhood acute lymphoblastic leukemia (ALL) [168]. Based on this discovery, a series of one-carbon-metabolism-targeted drugs have been developed, including methotrexate, pemetrexed, and 5-FU, which are of great significance in cancer treatment, especially immunotherapy [93, 169–177]. Ly345889 is the first synthetic inhibitor of MTHFD1/2 [148]. Subsequently, researchers found that carolacton, which is a macrolide ketone carbonic acid, inhibits folic acid-dependent one-carbon metabolism by targeting MTHFD1/2, and its inhibitory activity is higher than that of Ly345889 [149]. DHFR and TYMS were the early enzymes of one-carbon metabolism to be clinically validated as targets for cancer therapy and remain the most successful in this context to date, and almost all Food and Drug Administration (FDA)-approved DHFR and TYMS inhibitors are classical or non-classical folate derivatives [178]. There are also several compounds targeting these two enzymes in the clinical trial pipeline: Trimetrexate [156], Raltitrexed [157], Piritrexim [158], ZD-9331 [159], GS7904L [160], and ONX-0801 [161]. Folate transporters play important roles in the efficacy of anti-folate chemotherapies [179]. Farletuzumab is a monoclonal antibody specifically targeting folate receptor beta (FOLR1) [180, 181]. Despite encouraging preclinical data, farletuzumab has not successfully completed the Phase III trial [180]. The biosynthesis of purines is carried out by purinosome and requires phosphoribosylglycinamide formyltransferase (GART) [182]. Lometrexol has the strongest inhibitory effect on GART, which is in clinical trials [163, 183]. Moreover, the methionine cycle is crucial for one-carbon metabolism because the one-carbon unit is transferred to S-adenosyl-l-homocysteine (SAH) to form SAM in this process, which is necessary for many life activities [184]. AG-270, targeting methionine adenosyltransferase 2 A (MAT2A) in the methionine cycle, is currently in a cinical trial (NCT03435250) [162]. Cystathionine beta-synthase (CBS) is the first rate-limiting enzyme in the transsulfuration pathway and play vital roles in the occurrence, development, and treatment of cancer [185]. As a classical inhibitor of CBS, aminooxyacetic acid is the most common, but it also inhibits cystathionine gamma-lyase (CSE), which is another enzyme in the transsulfuration pathway [164].

In summary, the investigation of inhibitors of SGOC metabolism will help to clarify the role of one-carbon metabolism in different stages of cancer progression and to verify whether one-carbon metabolism is the right pathway to drugs for cancer treatment. The newer generation of drugs selectively targeting key metabolic enzymes in SGOC metabolism, such as PHGDH, MTHFDs, DHFR, TYMS, GART and CBS, will provide a new strategy for cancer treatment in the future.

## Conclusion and prospects

More than 70 years ago, Faber and his colleagues found that folic acid can stimulate the proliferation of acute lymphoblastic leukemia cells, and used aminopterin to induce clinical remission in patients [168]. At later time periods, more drugs targeting the one-carbon metabolic pathway were found, such as the folic acid metabolism and thymidine acid synthesis inhibitors: methotrexate and 5-Fu, which are among the first successful cancer treatments [186]. These drugs are still used to treat various cancers. When the PHGDH-specific inhibitor NCT-503 or shRNA was used to inhibit PHGDH expression, the antitumor effect of doxorubicin in TNBC was significantly improved in vivo and in vitro [187]. In addition, pemetrexed could increase the activation of T cells in mouse tumors in vivo and exerted the inherent effect of T cells in vitro; moreover, combined with PD-1 pathway blockade, pemetrexed enhanced the anti-tumor effect [93]. These evidences indicate that the inhibition of specific enzymes in SGOC metabolism can be a more effective mechanism for synergistic treatment of drug resistance and enhancing tumor immunotherapy.

To generate enough one-carbon unit to meet their own proliferation needs, tumor cells usually increase their intake of extracellular serine, glycine and other raw materials [48, 52]. Therefore, limitations of serine and glycine in the diet may be a good treatment strategy [21]. Tumors with amplified SGOC metabolic enzymes are unlikely to be affected by exogenous serine consumption, and p53 deletion may aggravate this dependence [20]. This scenario may be due to the impaired transformation between glycolysis and oxidative phosphorylation when p53 is deficient, thus resulting in insufficient ATP production [22]. It has been found that the growth rate of tumors and the final volume of tumors in mice treated with a serine- or glycine-deficient diet combined with metformin significantly decreased [188]. The mechanism may be that serine deficiency can inhibit the compensatory increase in the metformin induced glycolysis pathway. Therefore, the identification of metabolic dependencies related to the environment may help to identify tumor types that may benefit from existing approved therapies and can be more easily reused for new applications [8, 189].

SGOC metabolism not only serves as precursors to protein synthesis, but also provides one-carbon precursors for nucleotide synthesis, as well as head groups for sphingolipid and phospholipid synthesis [18, 44]. It seems that many cancer cells depend on the availability of one-carbon unit to some extent, which indicates that the limitation of one-carbon unit supply can have more therapeutic benefits. This review comprehensively analyzes the expression pattern and metabolic flux of the SGOC pathway in multiple cancer backgrounds at the system level and to describe the possible role of the SGOC metabolic network in tumor immunotherapy and ferroptosis. It is expected that new research and more effective and specific compounds may provide much-needed breakthroughs in targeting this pathway against cancer.

## Data Availability

Not applicable.

## Abbreviations

α-KG: α-ketoglutarate
ADP: Adenosine diphosphate
ALDH1L1/2: Aldehyde dehydrogenase 1 family member L1/L2
AML: Acute myeloid leukemia
AMPK: AMP activated protein kinase
ATP: Adenosine triphosphate
BHMT: betaine-homocysteine S-methyltransferase
B12: Vitamin B12
B6: Vitamin B6
CBS: Cystathionine beta-synthase
CDNs: Cyclic dinucleotides
CH^+^-THF: 5,10-methenyl-THF
CH_2_-THF: 5,10-methylenetetrahydrofolate
CMPSA: p-chloromercuriphenylsulfonic acid
CRC: Colorectal cancer
CSE: Cystathionine gamma-lyase
DHF: Dihydrofuran
DHFR: Dihydrofolate reductase
DMG: Dimethylglycine
EMT: Epithelial-mesenchymal transition
ESCC: Esophageal squamous cell carcinoma
FDA: Food and Drug Administration
FOCM: Folate-mediated one-carbon metabolism
FOLR1: Folate Receptor Alpha
FRGs: Ferroptosis-related genes
FRβ: Folate receptor beta
GCSCs: Gastric cancer stem cells
GART: Phosphoribosylglycinamide formyltransferase
GCLC: Gamma-glutamyl cysteine synthetase
GGCT: Gamma-Glutamylcyclotransferase
GLDC: Glycine dehydrogenase
GLS: Glutaminase
Glu: Glutamate
GOT1: Glutamate oxaloacetate transaminase
Gpx2: Glutathione peroxidase 2
GSH: Glutathione
GSS: Glutathione synthetase
H3K9me1: Histone H3 lysine 9 monomethylation
H3K9me3: Histone H3 lysine 9 trimethylation
HCC: Hepatocellular carcinoma
HNSCC: Head and neck squamous cell carcinoma
IGF2: Insulin-like growth factor 2
ILF3: Interleukin enhancer binding factor 3
LncRNAs: Long noncoding RNAs
MAT: Methionine adenosyltransferase
MAT2A: Methionine adenosyltransferase 2 A
MS: Methionine synthase
MTHF: Methylenetetrahydrofolate
MTHFD1/1L: Methylenetetrahydrofolate dehydrogenase 1/1L
MTHFD2/2L: Methylenetetrahydrofolate dehydrogenase 2/2L
MTHFR: Methylenetetrahydrofolate reductase
NADPH: Nicotinamide adenine dinucleotide phosphate
NSC127755: Trichloroethylene anti folic acid
NSCLC: Non-small cell lung cancer
PCNSL: Primary central nervous system lymphoma
PEP: Phosphoenolpyruvate
PHGDH: Phosphoglycerate dehydrogenase
PSAT1: Phosphoserine aminotransferase 1
PSPH: Phosphoserine phosphatase
ROS: Reactive oxygen species
SAH: S-adenosyl-l-homocysteine
SAM: S-adenosyl-l-methionine
SGOC: Serine-glycine-one-carbon
SHMT1/2: Serine hydroxymethyltransferase 1/2
SUMOs: Small ubiquitin-like modifiers
THF: Tetrahydrofolate
TYMS: Thymidylate synthase
2PG: 2-phosphoglycerate
3PG: 3-phosphoglycerate
3PH: 3-phosphohydroxypyruvate
3PS: 3-phosphoserine
10-CHO-THF: 10-formyltetrahydrofolate
5-Fu: 5-fluorouracil
5-CH_3_-THF: 5-methyltetrahydrofolate
